# Cytoreductive Surgery Under Aminolevulinic Acid-Mediated Photodynamic Diagnosis Plus Hyperthermic Intraperitoneal Chemotherapy in Patients with Peritoneal Carcinomatosis from Ovarian Cancer and Primary Peritoneal Carcinoma: Results of a Phase I Trial

**DOI:** 10.1245/s10434-014-3901-5

**Published:** 2014-07-24

**Authors:** Yang Liu, Yoshio Endo, Takuji Fujita, Haruaki Ishibashi, Toshihiro Nishioka, Emel Canbay, Yan Li, Shun-ichiro Ogura, Yutaka Yonemura

**Affiliations:** 1 NPO to Support Peritoneal Dissemination Treatment, 1-26 Haruki-Moto-Machi, Osaka, 596-0032 Japan; 2 Peritoneal Dissemination Center, Kishiwada Tokushukai Hospital, Kishiwada, Japan; 3 Cancer Research Institute, Kanazawa University, Kanazawa, Japan; 4 Department of Oncology, Zhongnan Hospital, Wuhan, China; 5 Frontier Research Center, Tokyo Institute of Technology, Yokohama, Japan

## Abstract

**Background:**

We conducted a phase I clinical trial to evaluate the sensitivity, specificity, and safety of cytoreductive surgery (CRS) under aminolevulinic acid-mediated photodynamic diagnosis (ALA-PDD) plus hyperthermic intraperitoneal chemotherapy (HIPEC) on 20 patients with peritoneal carcinomatosis (PC) from ovarian cancer and primary peritoneal carcinoma (PPC).

**Patients and Methods:**

Patients took 5-aminolevulinic acid (5-ALA) at a dose of 20 mg/kg orally with 50 mL of water 2 h before surgery. During surgery, the abdominal cavity was observed under blue light (wavelength of 440 nm) before and after CRS plus HIPEC. Specimens were excised and submitted for pathological examination to evaluate the specificity of ALA-PDD. Postoperative course was closely monitored and detailed information was recorded.

**Results:**

CRS under ALA-PDD plus HIPEC was performed 21 times in 20 patients with PC (16 ovarian cancer, 4 PPC) between June 2011 and October 2013. With the exception of 1 (5 %) patient, strong red fluorescence was detected in 19 patients with ovarian cancer, with a sensitivity of 95 %. All specimens from red fluorescent lesions were invaded by cancer cells, with a specificity of 100 %. No severe adverse events occurred during the perioperative period, with the exception of some abnormal laboratory results and mild complications. All patients were alive until the last follow-up.

**Conclusion:**

ALA-PDD provided a high sensitivity and specificity in detecting peritoneal metastasis in patients with PC from ovarian serous carcinoma and PPC. CRS under ALA-PDD plus HIPEC was a feasible and safe treatment option for patients with PC from ovarian cancer and PPC.

Ovarian cancer is the eighth most frequent cancer in women, and the main cause of death from gynecological cancers worldwide.^1^ In Japan, there are about 10,000 new cases and 4,600 deaths annually.^2^ The majority of patients were in the advanced stage at first diagnosis, with a 5-year survival rate of 15–37 %.^3^ Primary peritoneal carcinoma (PPC) is a rare extra-ovary serous malignancy originating from the peritoneum, which has similar histological and clinical characteristics as advanced ovarian cancer.^4^ Advanced ovarian cancer and PPC progress principally within the abdominal cavity with ascites, lymph node metastasis, and peritoneal dissemination. A new strategy with a combination of cytoreductive surgery (CRS) and hyperthermic intraperitoneal chemotherapy (HIPEC) has been widely demonstrated as being effective for treating patients with peritoneal carcinomatosis (PC).^5^–^9^ Selected patients with advanced ovarian cancer and PPC could also benefit from optimal CRS and HIPEC.^4^,^10^

Aminolevulinic acid-mediated photodynamic diagnosis (ALA-PDD) is a new and effective method of detecting dysplasia and early-stage cancer lesion. It works by detecting a specific wavelength light generated from cancer cells under irradiation with an exciting light after administration of 5-aminolevulinic acid (5-ALA). ALA is a prodrug of heme biosynthesis. It can be converted to the photosensitizing agent protoporphyrin IX (PpIX). After administration of 5-ALA, PpIX accumulates in cancer tissue in a tumor-specific form, which contributes to easy identification of cancer tissue through irradiation with an exciting light. Meanwhile, cancer cells also get killed by reactive oxygen species, a cytotoxic production produced by PpIX under irradiation of exciting light. This strategy is named ALA-mediated photodynamic therapy (ALA-PDT).^11^ Due to their tumor-specific characteristics and less invasion, ALA-PDD and ALA-PDT have been widely applied in detecting and treating a variety of dysplasias and cancers such as Barrett’s esophagus, ulcerative colitis, skin cancer, brain cancer, gastric cancer, bladder cancer, and ovarian cancer.^11^–^13^

According to the study of Löning et al.^14^, ALA-PDD was able to provide high sensitivity for the detection of peritoneal disseminated tumor for laparoscopic diagnosis in patients with ovarian cancer after intraperitoneal administration of 5-ALA. This might be helpful for treating patients with PC from ovarian cancer and PPC by CRS plus HIPEC. Therefore, we performed a phase I trial to evaluate the feasibility and safety of CRS under ALA-PDD plus HIPEC in patients with PC from ovarian cancer and PPC.

## Patients and Methods

### Patients

From June 2011 to October 2013, a phase I clinical trial for treating patients with PC from ovarian cancer and PPC by CRS under ALA-PDD plus HIPEC was conducted in the Peritoneal Dissemination Center of Kishiwada Tokushukai Hospital, Osaka, Japan. The protocol of this study was approved by the Ethics Committee of Kishiwada Tokushukai Hospital (No-24-05), and all patients provided written informed consent to participate in this study. A total of 20 patients were enrolled in the study—16 patients with ovarian cancer, and 4 patients with PPC. The major clinicopathological characteristics of the patients are listed in Table 1.

**Table 1:** Clinicopathological characteristics of 20 patients with peritoneal carcinomatosis

Characteristics	Value
Demographic parameters	
Age (years, range [median])	44–75 (63)
Sex (female/male)	19/1
Clinicopathological parameters (*n*)	
Ovarian cancer	16
Primary peritoneal carcinoma	4
Histological diagnosis	
Serous adenocarcinoma	16
Serous papillary carcinoma	3
Serous papillary adenocarcinoma	1
Peritoneal carcinomatosis index (range)	2–33
Completeness of cytoreduction (range)	0–3

### Cytoreductive Surgery Under ALA-PPD Plus Hyperthermic Intraperitoneal Chemotherapy

All patients were treated with the intention of achieving complete CRS. Meticulous preoperative assessment was performed. Patients were administered 5-ALA (Cosmo Bio Co., Ltd, Tokyo, Japan) at a dose of 20 mg/kg orally with 50 mL of water 2 h before surgery, which was performed under general anesthesia and hemodynamic monitoring. Once the abdominal wall was open, 20 mL of ascites was sent for cytological examination. Meanwhile, detailed evaluation of the peritoneal cancer index (PCI) was conducted according to the principle of Sugarbaker.^15^ After evaluation, four biopsy specimens were excised, two of which were tumor tissue and two were normal tissue. Tumor tissue and normal tissue were discriminated under white light. Next, all lights in the surgery room were turned off. The abdominal cavity was observed under blue light (wavelength of 440 nm). Tumor tissue would emit red fluorescence under irradiation of blue light. Another four biopsy specimens were then taken—two specimens from red fluorescent lesions and two from non-fluorescent areas. All biopsy specimens were submitted for pathological examination. Optimal CRS was then carried out according to the peritonectomy procedure described by Sugarbaker.^15^

HIPEC was performed after optimal CRS with an open technique for 40 min, with 4 L of heated saline containing 40 mg of docetaxel (Sanofi K.K. Co., Ltd, Tokyo, Japan) and 100 mg of cisplatin (Nippon Kayaku Co., Ltd, Tokyo, Japan). The temperature of the solution was maintained at 42.9–43.5 °C during the entire procedure. After HIPEC, the abdominal cavity was again observed under blue light to check if the tumors had been totally removed. Drainage tubes were placed at appropriate sites and chest drainage tubes were also placed if subphrenic peritonectomy was performed. The wound was closed, and the patient was sent to the recovery room.

The extent of CRS was determined by completeness of cytoreduction (CC), according to the criteria described by Sugarbaker.^15^ CC-0 indicates no residual tumor; CC-1 represents <2.5 mm of residual tumor; CC-2 indicates residual tumor between 2.5 mm and 2.5 cm; and CC-3 indicates >2.5 cm of residual tumor.

### Postoperative Monitoring and Follow-Up

All patients were closely monitored for the following parameters: vital signs, drainage, flatus passage, and any discharge. Complete peripheral blood tests and blood chemistry were examined on the first, fourth, and seventh postoperative day. Pulmonary cardiovascular functions were monitored. Other postoperative care information was also detailed and recorded, including condition of the incision, time on liquid food, time to suture removal, time to remove drainage tube, and time to be discharged. Three weeks after being discharged, patients received intraperitoneal, intravenous, or oral chemotherapy. Adverse events that occurred during the perioperative period were graded according to the NCI-CTC Version 4.

All patients were routinely followed up either by outpatient clinic or telephone. The last time of follow-up was 1 February 2014.

### Statistical Analysis

Data were obtained from a database of clinical records, surgical reports, laboratory and pathology reports, and follow-up records. The numerical data were directly recorded, and the category data were recorded into different categories. The survival time was calculated from the date of first CRS under ALA-PDD plus HIPEC to the date of patient death. Data were analyzed by SPSS software, version 17.0 (SPSS^®^, Inc., Chicago, IL, USA), with a *p* value <0.05 considered to be statistically significant.

## Results

CRS under ALA-PDD plus HIPEC was performed 21 times in 20 patients. One patient with ovarian cancer underwent another CRS under ALA-PDD plus HIPEC for a second look 18 months after the first operation. For all 21 surgeries, all patients underwent optimal CRS. From the results of cytological examination, there were 12 episodes where cancer cells were found to be positive. The PCI of patients was 2–33 (median 11). Complete cytoreduction of CC-0/CC-1 was achieved 14 times. Incomplete cytoreduction of CC-2/CC-3 was performed seven times. Time of surgery ranged from approximately 1.5–6.0 h (median 4.0 h). The volume of blood loss during surgery was 300–2,400 mL (mean 950 mL). Mean blood requirement was 6.4 (range 0–10) units of packed red cells and for fresh frozen plasma was 8.6 (range 2–10) units.

### Postoperative Course

Postoperative vital signs of all patients were almost in the normal range and stable within the first postoperative week, except one patient who experienced a heart rate of >100 times per min after surgery and two patients who experienced fever with a temperature of 38.0 °C. No hypertension or hypotension occurred during the perioperative course. Laboratory results of the peripheral blood test, liver function, renal function, and C-reactive protein (CRP) on the first and seventh postoperative day, and on the day of discharge, are listed in Table 2. On the first postoperative day, the most common abnormalities were hypoproteinemia, high white blood cell count, and alternation of liver function. One week later, CRP remained abnormal in the majority of patients, but decreased continually to reach normal levels. On the day of discharge, almost all data were in the normal range. The incisions recovered well without any infection. The time to suture removal ranged from 7 to 13 days (median 10 days), time on liquid food ranged from 3 to 12 days (median 5 days), time to remove the drainage tube ranged from 3 to 7 days (median 4 days), and time to be discharged ranged from 10 to 42 days (median 28 days).

**Table 2:** Laboratory results of postoperative course

Parameter	Range (median)			Normal value
	Day 1	Day 7	Day of discharge	
Hg (g/dL)	8.0–12.6 (10.0)	10.2–11.3 (11.0)	10.6–12.6 (11.6)	12.0–16.0
RBC (10^4^/μL)	246–402 (350)	327–368 (347)	342–423 (370)	360–480
WBC (10^2^/μL)	55–161 (122.5)	70–110 (92)	51–81 (60)	40–85
NEU %	64.5–88.0 (83.8)	72.0–80.6 (76.5)	43.2–75.0 (65)	40–70
Platelet (10^4^/μL)	9.9–41.9 (13.5)	23.9–35.3 (27.5)	15.0–42.0 (26.0)	13.0–36.0
AST	32–129 (80)	20–35 (29)	17–37 (24)	13–37
ALT	18–127 (101)	8–52 (13)	8–45 (25.5)	8–45
TB	0.53–2.04 (1.14)	0.49–1.22 (0.84)	0.38–1.25 (0.89)	0.30–1.30
ALB	2.4–3.1 (2.7)	3.2–3.9 (3.6)	3.4–4.8 (4.1)	4.1–5.2
BUN	6.6–18.1 (10.3)	6.5–17.9 (11.9)	6.8–18.3 (13.0)	7.8–18.9
Creatine (U/L)	0.45–1.32 (0.63)	0.48–0.93 (0.55)	0.56–0.81 (0.67)	0.45–0.82
CRP	0.4–10.2 (6.24)	0.86–9.30 (4.80)	0.10–2.02 (1.20)	0.00–0.30

*Hg* hemoglobin, *RBC* red blood cell, *WBC* white blood cell, *NEU* neutrophilic granulocyte, *AST* aspartate aminotransferase, *ALT* alanine aminotransferase, *TB* total bilirubin, *ALB* albumin, *BUN* blood urea nitrogen, *CRP* C-reactive protein

### Adverse Events and Follow-Up

No severe adverse event occurred in the perioperative period; however, one patient lost her appetite for 12 days after the operation, one patient experienced diarrhea for 3 days, and two patients were with pleural effusion after subphrenic peritonectomy. All these symptoms disappeared after taking timely treatment. By the last follow-up on 1 February 2014, all 20 patients were alive. Detailed information for each patient is summarized in Table 3.

**Table 3:** Clinical and pathological features of 20 patients undergoing 21 episodes of CRS under ALA-PDD plus HIPEC

Patient no.	Age (years)	Sex	Diagnosis	Histology	Fluorescence emission	PCI	CC	Survival (months)
1	64	F	OC	Serous adenocarcinoma	Yes	30	3	4
2	60	F	OC	Serous adenocarcinoma	Yes	27	2	12
3	63	F	OC	Serous adenocarcinoma	Yes	5	0	22
4	44	F	OC	Serous adenocarcinoma	Yes	11	0	24
5	48	F	OC	Serous adenocarcinoma	Yes	3	0	18
6	65	F	OC	Serous adenocarcinoma	Yes	12	1	8
7	66	F	OC	Serous adenocarcinoma	Yes	6	0	10
8	66	F	OC	Serous adenocarcinoma	Yes	12	3	6
9	67	F	OC	Serous adenocarcinoma	Yes	18	1	12
10	65	F	OC	Serous adenocarcinoma	Yes	25	2	10
11	64	F	OC	Serous adenocarcinoma	Yes	2	0	20
12	75	F	OC	Serous adenocarcinoma	Yes	15	0	16
13	49	F	OC	Serous adenocarcinoma	Yes	4	0	15
14	60	F	OC	Serous adenocarcinoma	No	4	0	12
15	68	F	OC	Serous adenocarcinoma	Yes	6	0	16
16	45	F	OC	Serous adenocarcinoma	No	35	2	27
16*	47	F	OC	No malignancy	Yes	2	0	27
17	59	F	PPC	Serous papillary carcinoma	Yes	8	0	26
18	61	F	PPC	Serous papillary carcinoma	Yes	24	2	17
19	63	M	PPC	Serous papillary carcinoma	Yes	30	3	24
20	61	F	PPC	Serous papillary adenocarcinoma	Yes	2	0	32

*CRS* cytoreductive surgery, *ALA-PDD* aminolevulinic acid-mediated photodynamic diagnosis, *HIPEC* hyperthermic intraperitoneal chemotherapy, *F* female, *M* male, *OC* ovarian cancer, *PPC* primary peritoneal carcinoma, *PCI* peritoneal cancer index, *CC* completeness of cytoreduction

16* the same patients as No 16, who underwent a second CRS under ALA-PDD plus HIPEC

### Sensitivity of ALA-PDD During Operation

**Fig. 1:**
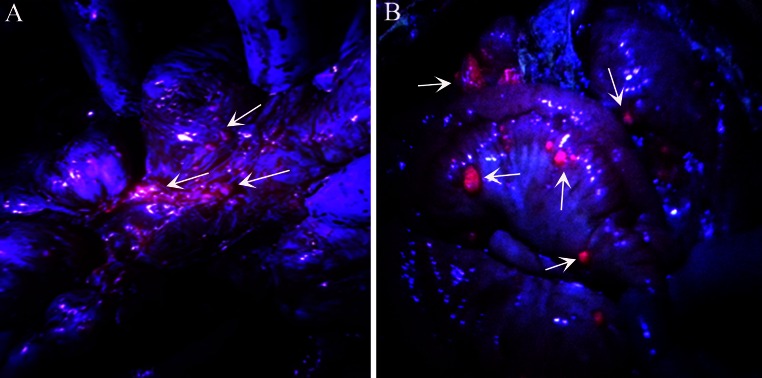
Peritoneal disseminated tumors from ovarian cancer and primary peritoneal carcinoma emitted strong red fluorescence under irradiation of blue light. *Arrows* indicate peritoneal disseminated tumor emitting strong red fluorescence. **a** Peritoneal disseminated tumors from ovarian cancer; **b** peritoneal disseminated tumors from primary peritoneal carcinoma

**Fig. 2:**
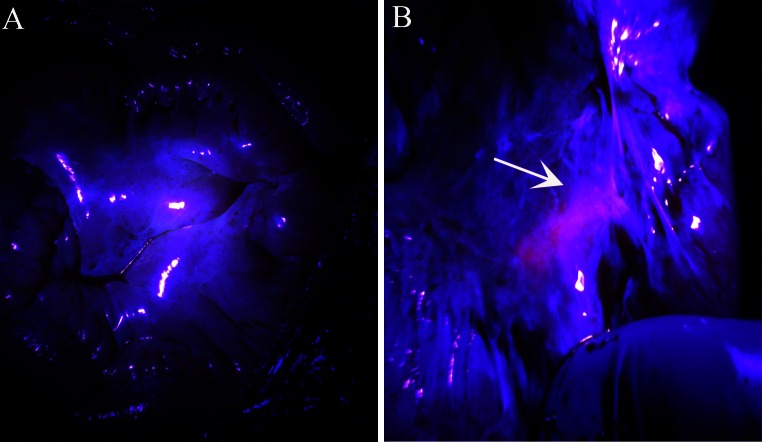
Recheck residual tumors under irradiation of blue light after cytoreductive surgery and hyperthermic intraperitoneal chemotherapy. **a** No red fluorescence was detected under irradiation of blue light after complete cytoreduction; **b** residual tumor on bowel mesentery still emitted weak red fluorescence after cytoreductive surgery and hyperthermic intraperitoneal chemotherapy

During 20 episodes of initial CRS under ALA-PDD plus HIPEC, peritoneal metastases emitted red fluorescence under irradiation of blue light in 19 patients, with a sensitivity of 95 % (Fig. 1), while peritoneal disseminated gross tumor nodules seen with white light in one (5 %) patient with ovarian cancer did not emit red fluorescence under irradiation of blue light. The smallest tumor that we detected was 0.5 mm in diameter. For the second surgery of the patient with ovarian cancer, no red fluorescence was detected, and no malignancy was found from the results of frozen and final pathological examination. Moreover, residual tumor in all patients undergoing incomplete CRS emitted red fluorescence after HIPEC (Fig. 2).

### Pathological Examination Results of Biopsy Specimens

For 21 surgeries, a total of 158 biopsy specimens were excised and examined. In the second surgery of CRS under ALA-PDD for the patient with ovarian cancer, no specimen was excised since no red fluorescence was detected, and frozen pathological examination showed no malignancy. All 40 tumor specimens obtained under white light and 38 specimens from red fluorescent lesions were positive for cancer cells, with a specificity of 100 %. Among 40 normal specimens excised under white light, five specimens were tumors. For the other 40 specimens from the non-fluorescent area, three specimens were tumors, with a false negative rate of 7.5 % (Table 4).

**Table 4:** Results of biopsies under white light and under irradiation of blue light

	Tumor	No tumor	*p* value
White light			
Tumor	40	0	0.000
Normal tissue	5	35	
Blue light			
Red fluorescence	38	0	0.000
No red fluorescence	3	37	

## Discussion

To our knowledge, this is the first study suggesting that ALA-PDD may be effective in detecting peritoneal metastasis originating from PPC, as well as from ovarian cancer. A series of experimental and clinical studies demonstrated that ALA-PDD was able to detect peritoneal metastases from different cancers.^14^,^16^–^18^ Löning et al.^14^ reported the first study evaluating the use of ALA-PDD in finding peritoneal metastasis of epithelian ovarian carcinoma. In their study, peritoneal metastases in 12 cases emitted strong red fluorescence among 13 patients after intraperitoneal application of 5-ALA. In our study, peritoneal metastases in 15 patients with PC from ovarian cancer emitted strong red fluorescence after oral application of 5-ALA. With a combination of the results of Löning et al.^14^. and our results, peritoneal metastases in 27 cases emitted strong red fluorescence among 29 patients treated by ALA-PDD, with a high sensitivity of 93.1 %. This may provide more convincing evidence to support the fact that ALA-PDD could provide a high sensitivity of detecting peritoneal metastasis in patients with ovarian cancer. Moreover, in all four cases of PPC, peritoneal metastases also emitted strong red fluorescence. Although four cases were not enough, they still provided evidence that ALA-PDD might be effective in detecting peritoneal metastasis in patients with PCC. In addition, we noticed that all patients in this study had serous adenocarcinoma or serous papillary carcinoma. These cancers share similar histological and clinical features. Therefore, we infer that ALA-PDD might also be sensitive in detecting peritoneal metastasis from other serous carcinoma.

This is also the first study evaluating the safety of CRS plus HIPEC with the application of ALA-PDD. It suggests that CRS under ALA-PDD plus HIPEC would be a safe strategy for treating patients with PC. Usually, ALA-PDD is a safe procedure; however, some adverse events have been reported. The main potential side effects of oral administration of 5-ALA included hypotension, alternation of liver function, urinary frequency, nausea, and vomiting.^19^,^20^ In addition, CRS plus HIPEC has a high risk of postoperative complications. As a result, it was necessary to evaluate safety first. In the present study, no severe adverse event occurred, with the exception of two patients with pleural effusion, one patient who lost her appetite, one patient who experienced diarrhea, and some abnormal results in laboratory data. It was difficult to prove a direct relationship between such complications and ALA-PDD. However, all these abnormalities disappeared after timely treatment and patients underwent uneventful recovery courses. This good result might be attributed to a well-experienced team with good team work and a safe dose of 20 mg/kg of 5-ALA, as recommended by Chung and Eljame.^19^

For patients with PC from ovarian cancer and PPC undergoing CRS and HIPEC, complete cytoreduction was an independent factor predicting better survival and prognosis.^4^,^21^ Therefore, tumors should be removed as far as possible. ALA-PDD was helpful in achieving this goal; in addition to high sensitivity, it could detect a microscopic tumor with high specificity. The smallest tumors detected by ALA-PDD had a diameter of 0.5 mm, in the present study. In another report, smaller tumors of <0.5 mm diameter were also detected.^14^ Thus, microscopic tumors that were usually ignored might be removed with the help of ALA-PDD. Moreover, Takahashi et al.^22^, found that administration of ALA markedly enhanced the antitumor effect of hyperthermotherapy on an animal mode; it might also enhance the effect of HIPEC . For this reason, we believe that ALA-PDD provided help not only on surgery, but also on chemotherapy.

Several studies on treating patients with PC by PDT have been performed; however, the results were not encouraging. In the study of Hendren et al.^23^, 42 patients received PDT under irradiation of laser after debulking surgery with a residual tumor ≤5 mm in diameter, with a median survival of 21 months. However, the study had some deficiencies: (1) lack of control results for patients undergoing only debulking surgery to prove the contribution of PDT and adverse effects related to PDT; (2) Photofrin used in their study could not produce enough cytotoxic effect to cancer cells, and, in addition, Photofrin required a long clearance time of 4–8 weeks after injection; and (3) exact efficacy of debulking surgery with PDT in patients with a specific type of cancer could not be told owing to ignoring different effects of PDT on various cancers. Similar problems were found in another similar study reported by Hahn et al.^24^ CRS plus HIPEC has been proven to have better efficacy than debulking surgery. Moreover, 5-ALA used in our study has been proven to be better than Photofrin in enhancing the effect of ALA-PDD. We believe that patients with PC originating from ovarian cancer and PPC would benefit more from the new treatment of CRS under ALA-PDD plus HIPEC.

## Conclusions

CRS under ALA-PDD plus HIPEC is a feasible and relative safe treatment option in selected patients with PC originating from ovarian cancer and PPC. With the results of this study, a higher-level clinical trial needs to be performed to provide more supportive evidence.
